# International accreditation and quality medical education; the need for establishment in middle east

**Published:** 2013-09-30

**Authors:** Afshin Khani, Amin Zarghami

**Affiliations:** 1 Research Committee, Babol University of Medical Science, Babol, Iran.

## Dear Editor,

We read with a great interest the recent article by Pulido PA^1^ that published in your journal in which he addressed the role of increasing number of medical schools on the efficacy of medical education and the training of futures doctors. In this article the author emphasized on the social accountability and the commitment of medical schools for reflect critically and enhancing the quality of their graduates. Finally he suggest an accreditation system with certain objectives including adjustment of medical education with modern technologies, preparing doctors for needs and expectations of a globalized society and educating lifelong learning skills to physicians.

 Here in Iran like other developing countries in the Middle East region, the need for enhancing health care system forced the government to increase the number of physicians to reach the appropriate patient to physician ratio. Hence, quick increase in the number of medical schools and excessive admitting of medical students implemented in Iran. While this trend leads to train lots of junior doctors across the country, the quality of their training program and their clinical competency seems to be neglected. On the other hand, much concern has been expressed about the caliber of many junior doctors from these various institutions. Moreover, due to overwhelming number of junior doctors in some parts of country, there is lack of appropriate clinical opportunities for junior doctors as General Physicians (GP) and the lack of clinical experience lead to distrust of general population toward GPs. Therefore, majority GP doctors are eager to study for residency course instead of doing their clinical duties. So, the depletions must be identified and appropriate changes made, for enhancing the quality and standards of medical education for undergraduates. Altogether, the need for an organization which evaluates the competency of graduates of medical schools is highly obvious in developing countries of our region.

 As the author underlined the essentiality of the issue of accreditation for institutes both for undergraduate and post graduate students by mentioning the*" lifetime of continuing education". *It is well known that traditional continuing medical education has a limited impact on their clinical behavior and the requirement for continuous learning as part of a doctor's professional career is obvious. However, the appropriate ways of introducing and implementing this learning is a matter of controversy in many countries even in a developing country, like Iran. Continuing medical education should meet the doctors' educational needs by presenting a variety of learning options to suit their personal learning styles. Unfortunately, physicians must accept responsibility for their own continuous learning to reach their intended goals. Thus, we need to find some effective methods in order to be addressed in a trusted way. Such objectives could be achieved and administered just by a superior institution or committee consists of professionals.

According to successful experience of the Pan American Federation of Faculties (Schools) of Medicine (FEPAFEM/PAFAMS) in the American continent, we undoubtedly support the belief that fundamental restructuring of medical education needed today. We suggest to establish such an association or federation in our region in order to achieve the same objectives of FEPAFEM's in Middle East countries that " *use evaluation and accreditation as part of a change management strategy, along with modernization and improvements to the quality of medical education on the to meet present and future challenges."*

## Responding to:

Dear Sir: Your note reflects the need of a reality check on your medical education efforts in Iran. Examining what you are d doing in Evaluation and proper accreditation of the Medical Education and Health System in Iran is an important first step. You may do this taking into considerations the realities of the region, thus, among others, the participation of the World Fede ration for Medical Education, WFME could give valuable orientation. Important to o contact Professor Ibrahim Alwan, currently the President of the Eastern Mediterranean Region, AMEEMR, and Dean of the Medical School in Rijadh, Saudi Arabia, alwani@ksau-hs.edu.sa who could provide the initial framework to your valluable endeavor. In addition the WFME is also a primary source of orientatio on to your efforts (Drs. Stefan Lindgren Stefan.lindgren@med.lu.se and DDavid Gordon gordoncph@googlemail.com). 

Kind regards, 

Pablo Pulido fepafempafams@fepafempafamss.org
